# Development of a Serotyping Scheme for *Streptococcus pasteurianus*: An Underreported Zoonotic Pathogen

**DOI:** 10.1155/tbed/6779233

**Published:** 2026-06-28

**Authors:** Shuoyue Wang, Xinchun Li, Chenxu Zheng, Jinlu Zhu, Anusak Kerdsin, Zongfu Wu

**Affiliations:** ^1^ MOE Joint International Research Laboratory of Animal Health and Food Safety, College of Veterinary Medicine, Nanjing Agricultural University, Nanjing, 210014, China, njau.edu.cn; ^2^ Key Lab of Animal Bacteriology, Ministry of Agriculture and Rural Affairs, Nanjing Agricultural University, Nanjing, 210014, China, njau.edu.cn; ^3^ WOAH Reference Lab for Swine Streptococcosis, Nanjing Agricultural University, Nanjing, 210014, China, njau.edu.cn; ^4^ Faculty of Public Health, Kasetsart University, Chalermphrakiat Sakon Nakhon Province Campus, Sakon Nakhon, 47000, Thailand, ku.ac.th; ^5^ Special Research Unit for Tropical Zoonoses and Antimicrobial Resistance Surveillance, Kasetsart University, Bangkok, 10900, Thailand, ku.ac.th

**Keywords:** capsular polysaccharides, serotype, *Streptococcus pasteurianus*

## Abstract

*Streptococcus pasteurianus* is an opportunistic and underreported zoonotic pathogen capable of causing severe infections in both humans and animals, and it is also an etiological agent of swine streptococcosis. Despite its clinical and veterinary relevance, no systematic serotyping scheme has previously been established for this species. In this study, six representative strains harboring distinct *cps* gene clusters were selected for antiserum production, and agglutination assays identified five serotypes, designated serotypes 1a, 1b, 2, 3, and 4. Based on serotype‐specific variation within the *cps* gene cluster, a multiplex PCR (mPCR) serotyping assay was subsequently developed and evaluated using 35 *S. pasteurianus* strains, including 14 pig isolates, 19 human isolates, and two cattle isolates. Serotype assignments obtained by mPCR were fully concordant with those determined by agglutination assays using rabbit antisera. In addition, transmission electron microscopy (TEM) revealed a visible capsular layer surrounding representative strains of each serotype. Collectively, these findings establish a robust serotyping framework for *S. pasteurianus*. The mPCR serotyping assay provides a valuable tool for future epidemiological studies, enhances diagnostic capability, and supports improved surveillance and control of *S. pasteurianus* infections.

## 1. Introduction

*Streptococcus pasteurianus* is an opportunistic zoonotic pathogen that poses a significant threat to both human and animal health [1–4]. In humans, particularly neonates, the elderly, and immunocompromised individuals, it can cause severe diseases such as meningitis, septicemia, and life‐threatening conditions like endocarditis [5–8]. As an opportunistic pathogen, *S. pasteurianus* infects at least nine animal species and can cause septicemia, meningitis, and fatal outcomes [2–4, 9]. Recent studies have identified *S. pasteurianus* as a newly recognized pathogen associated with swine streptococcosis [9, 10]. This bacterium, frequently detected together with *Streptococcus suis*, has been isolated not only from diseased pigs but also detected in the hilar lymph nodes and tonsils of healthy pigs, indicating that asymptomatic carriers may serve as potential reservoirs for transmission [11]. In our previous study, we reported that a cocolonization rate of *S. pasteurianus* and *S. suis* was 9.79% in healthy pigs in China [10]. These findings highlight the veterinary and public health significance of *S. pasteurianus* and suggest that pigs may serve as an important reservoir for its transmission.

The capsule is a critical surface component in many bacterial species and serves as a major virulence factor in several important pathogens [12]. Our previous study confirmed that the capsule also plays an essential role in the pathogenesis of *S. pasteurianus* [9]. Capsule diversity arises primarily from differences in glycosyl composition and glycosidic linkages, which underlie antigenic variation and provide the molecular basis for serotype classification [12, 13]. In most bacteria, genes responsible for capsular polysaccharide (CPS) synthesis are organized into clusters within the chromosomal *cps* locus [14]. Therefore, analysis of the *cps* cluster has served as a fundamental approach in serotyping studies. For instance, *wzy* gene‐based typing is commonly used in *S. suis* research [15], whereas sequencing of the *cpsB*, *wzy*, and *wzx* has been used for initial serotype assignment in *Streptococcus pneumoniae* [12]. These molecular approaches can overcome several technical limitations of traditional serotyping, including the limited availability of specific antisera, cross‐reactivity, and autoagglutination. They also contribute to the identification of novel serotypes or genotypes and facilitate the analysis of genetic variation within the *cps* cluster. Among streptococci, distinct serotypes often differ substantially in tissue tropism and pathogenic potential [16, 17]. However, despite its association with severe infections in humans and animals, *S. pasteurianus* currently lacks serotype information and a systematic serotyping scheme, which limits a deeper understanding of its epidemiological characteristics and pathogenic mechanisms. Therefore, establishing a reliable serotyping method for *S. pasteurianus* is essential.

In this study, comparative analysis of the *cps* gene clusters from 17 *S. pasteurianus* genomes, together with agglutination assays using rabbit antisera, enabled the classification of these strains into five distinct serotypes. The presence of the capsular material was further confirmed by transmission electron microscopy (TEM). Based on serotype‐specific variation within the *cps* locus, we developed a multiplex PCR (mPCR) serotyping method, providing a practical tool for the rapid and accurate serotyping of *S. pasteurianus*. This method will facilitate future epidemiological surveillance, pathogenesis studies, and vaccine development for this pathogen.

## 2. Materials and Methods

### 2.1. Bacterial Strains and Culture Conditions

A total of 66 *S. pasteurianus* strains and genomes were included in this study. The strain collection comprised three distinct datasets. The first dataset consisted of 17 isolates, including 15 isolates from China, strain NCTC13784 obtained from the Culture Collection, University of Gothenburg, and strain ATCC 43144 from the American Type Culture Collection (Table 1). The second dataset included 18 isolates recovered from human and cattle sources in Thailand between 2022 and 2023 (Table 1). The third dataset comprised 31 publicly available *S. pasteurianus* genome sequences retrieved from the NCBI database (Supporting Information Table S1). These genomes originated from seven countries, including the United States (*n* = 10), China (*n* = 13), Denmark (*n* = 3), and one isolate each from Belgium, South Korea, Kenya, and Bangladesh; the country of origin was unknown for one genome.

**Table 1:** The information of strains used in this study.

Serotype	Reference strain^a^	Accession number	Country	Year	Isolation site	Host	Health status	Source
1a	**NCTC13784**	NZ_LS483462.1	France	Unknown	Cerebrospinal fluid	Human	Diseased	Culture Collection University of Gothenburg
	MN120	—	Thailand	2023	Blood	Human	Diseased	This study
1b	**WUSP067**	NZ_CP039457.1	China	2016	Brain	Pig	Diseased	This study
	WUSP068	NZ_JAWKHC000000000.1	China	2016	Brain	Pig	Diseased	This study
	WUSP069	NZ_JAWKHB000000000.1	China	2016	Brain	Pig	Diseased	This study
	WUSP084	NZ_JAWKHA000000000.1	China	2016	Brain	Pig	Diseased	This study
	MN93	—	Thailand	2022	Blood	Human	Diseased	This study
	MN104	—	Thailand	2023	Blood	Human	Diseased	This study
	MN121	—	Thailand	2023	Ascites	Human	Diseased	This study
	MN177	—	Thailand	2023	Blood	Human	Diseased	This study
	MN231	—	Thailand	2023	Blood	Human	Diseased	This study
	MN46	—	Thailand	2022	Blood	Human	Diseased	This study
2	**WUSP070**	NZ_CP116957.1	China	2021	Feces	Cattle	Diseased	This study
	SKF5.1	—	Thailand	2022	Unknown	Cattle	Unknown	This study
3	**ATCC43144**	NC_015600.1	Unknown	Unknown	Blood	Human	Diseased	American Type Culture Collection
	WUSP083	NZ_JAWKGW000000000.1	China	2023	Tonsil	Pig	Healthy	This study
	WUSP085	NZ_JAWKGV000000000.1	China	2023	Tonsil	Pig	Healthy	This study
	WUSP086	NZ_JAWKGU000000000.1	China	2023	Tonsil	Pig	Healthy	This study
	WUSP087	NZ_JAWKGT000000000.1	China	2023	Tonsil	Pig	Healthy	This study
	WUSP088	NZ_JAWKGS000000000.1	China	2023	Tonsil	Pig	Healthy	This study
	MN141	—	Thailand	2023	Unknown	Human	Diseased	This study
	MN176	—	Thailand	2023	Blood	Human	Diseased	This study
	MN198	—	Thailand	2023	Blood	Human	Diseased	This study
	STC1034	—	Thailand	2022	Blood	Human	Diseased	This study
	STC1385	—	Thailand	2022	Unknown	Human	Diseased	This study
	STC1394	—	Thailand	2022	Blood	Human	Diseased	This study
	STC1049	—	Thailand	2022	Blood	Human	Diseased	This study
4	**WUSP082**	NZ_CP136944.1	China	2023	Tonsil	Pig	Healthy	This study
	WUSP074	NZ_CP116958.1	China	2021	Tonsil	Pig	Healthy	This study
	WUSP079	NZ_JAWKGZ000000000.1	China	2021	Tonsil	Pig	Healthy	This study
	WUSP080	NZ_JAWKGY000000000.1	China	2021	Tonsil	Pig	Healthy	This study
	WUSP081	NZ_JAWKGX000000000.1	China	2021	Tonsil	Pig	Healthy	This study
	MN106	—	Thailand	2023	Blood	Human	Diseased	This study
	STC126	—	Thailand	2023	Blood	Human	Diseased	This study
	STC142	—	Thailand	2023	Blood	Human	Diseased	This study

^a^The bold text indicates the serotype reference strains of *S*. *pasteurianus*.

All 35 isolates were identified as *S. pasteurianus* using a mPCR assay targeting homologs of genes *E8M05_RS04035*, *E8M05_RS05155*, and *E8M05_RS06300* from the reference strain WUSP067, as described previously [10]. For the 31 publicly available genomes retrieved from NCBI, species identity was confirmed by the presence of these *S. pasteurianus*‐specific marker genes in the genome sequences. Bacterial strains were cultured in Todd–Hewitt broth (THB; Hope Bio‐Technology Co., Ltd., China) and plated on THB agar supplemented with 5% (v/v) sheep blood, followed by incubation at 37°C under 5% CO_2_.

### 2.2. Analysis of the Capsular Gene Cluster

It is well‐established that in most bacteria, the *cps* cluster is flanked by conserved genes: an upstream anchor gene typically involved in sugar‐nucleotide precursor biosynthesis and a downstream housekeeping gene often related to amino acid metabolism. In our study of *S. pasteurianus*, we identified a gene encoding a purine nucleoside phosphorylase (*deoD*) involved in sugar‐nucleotide precursor synthesis as a conserved gene located in the upstream of the *cps* locus. A conserved downstream gene of ~1200 bp with an unknown function was also detected. The *cps* gene cluster was analyzed using Prokka (v1.12) and Easyfig (v2.2.3). For *cps* cluster‐based grouping, strains exhibiting >90% sequence similarity were assigned to the same group.

### 2.3. Preparation of Rabbit Antiserum

Based on NCBI BLAST analysis of the *cps* gene clusters in *S. pasteurianus*, six representative strains, NCTC13784, WUSP067, WUSP070, ATCC43144, WUSP074, and WUSP082, were selected for this study; each strain exemplifies a distinct *cps* cluster type. To ensure vigorous growth and stable capsular expression, the selected strains underwent five serial passages in a liquid THB medium. Bacteria were inactivated by treatment with 0.5% formaldehyde under constant agitation at room temperature overnight. The inactivated cells were thoroughly washed with phosphate‐buffered saline (PBS) buffer and resuspended to appropriate concentrations for immunization. Chinese white rabbits were immunized by an intravenous injection through the marginal ear vein. Each rabbit received 1 mL of inactivated bacterial suspension according to the following schedule: 2–4 × 10^9^ colony‐forming unit (CFU) per dose on days 1, 14, 16, and 19, followed by 4–8 × 10^9^ CFU per dose on days 22, 28, 31, 34, 37, and 40 [18]. Collecting blood was performed aseptically 10 days after the final immunization. Blood samples were incubated at 37°C for 1 h, followed by overnight storage at 4°C, after which antisera were collected by centrifugation.

### 2.4. Agglutination Assay

To assess the antiserum titer following immunization with various strains, the slide agglutination test was utilized. The test antiserum was serially diluted using a diluent composed of 0.5% phenol in 0.85% NaCl to achieve dilutions of 1:2, 1:4, 1:8, 1:16, 1:32, 1:64, 1:128, 1:256, and 1:512. Nonimmune rabbit serum diluted 1:4 and PBS were used as negative controls. Ten microliters of each diluted antiserum were mixed with 10 μL of the prepared antigen (4 × 10^9^ CFU/mL) on a clean slide and thoroughly mixed. The mixture was observed for agglutination between 1 and 4 min at room temperature. The highest antiserum dilution exhibiting a positive agglutination reaction was recorded as the antiserum titer, with a titer below 1:4 recorded as negative for that specific serotype. Serotypes were ultimately defined by the combination of *cps* cluster grouping and unique agglutination patterns without cross‐reactivity.

### 2.5. Preparation of Absorbed Antisera

Absorbed antisera were prepared by incubating each rabbit antiserum with inactivated cells of the corresponding absorbing strain at a concentration of 200 mg/mL (wet weight) overnight at 4°C, as previously described [19]. Following incubation, the mixtures were centrifuged at 13,000×*g* for 2 min, and the resulting supernatants were collected and designated as the absorbed antisera. The absorption procedure was repeated until the agglutination titer of the antiserum against the corresponding inactivated cells decreased to below 1:2.

### 2.6. TEM

The TEM analysis was conducted to examine the capsular characteristics of *S. pasteurianus* strains NCTC13784, ATCC43144, WUSP070, and WUSP082, following a method similar to a previous study [9]. Briefly, bacterial cells from mid‐logarithmic phase cultures were collected by centrifugation and fixed with 2.5% glutaraldehyde in 0.1 M PBS (pH 7.4) for 4 h at 4°C. After washing with the same buffer, the samples were post‐fixed in 1% osmium tetroxide for 2 h, dehydrated through a graded ethanol series, and embedded in Spurr’s resin. Ultrathin sections (70 nm) were prepared using an ultramicrotome, stained with uranyl acetate and lead citrate, and observed under a Hitachi HT‐7700 transmission electron microscope operated at 80 kV.

### 2.7. mPCR Assay

To identify serotype‐specific targets for molecular serotyping, the *cps* gene clusters representing different serotypes were comparatively analyzed. Genes sharing less than 80% sequence similarity among serotypes (Supporting Information Table S2) were considered putatively serotype‐specific and selected as candidate targets. The targeted genes and primers are shown in Table 2. The optimal reaction system for the mPCR assay is as follows: 12.5 µL of 2× Rapid Taq Master Mix (Vazyme, Nanjing, China), 0.4 µL of 1a‐F/R, 1.5 µL of 1b‐F/R, 0.6 µL of 2‐F/R, 0.15 µL of 3‐F/R, 0.3 µL of 4‐F/R, 5.6 µL of ddH_2_O, and 1 µL of the template (each primer at a final concentration of 10 µM). PCR was performed with an annealing temperature of 52.0°C and an extension time of 40 s.

**Table 2:** Primer sequences used for mPCR.

Serotype	Target gene (locus tag)	Primer name	Primer sequence (5′−3′)	Product size (bp)
1a	*cpsH* (*DQN56_RS05055*)	1a‐F	TTGATGCAAATGGCTGAAGCAGAT	238
		1a‐R	CAGACGTACAACTAGCTGATGTAA	
1b	*cpsH* (*E8M05_RS04790*)	1b‐F	GGCAGAATCAGAACTGTTAACT	380
		1b‐R	TATTAATCTCAATAGCATCACCGGAT	
2	*cpsJ* (*M0P24_RS05765*)	2‐F	ATTGAGGGCGTAGCTGAATTTAGA	834
		2‐R	CTTCAGAAAAAGAAGAGCTATGGA	
3	*cpsI* (*SGPB_RS04335*)	3‐F	TTGACTGGTCTTCTAGGGGTATGG	544
		3‐R	CTGGGGTAAAAGTCAACTCTGCAA	
4	*cpsO* (*RJD36_RS07025*)	4‐F	GTGGAGGACCTGTAACGAGTATTA	1066
		4‐R	CTCAAACATAGCTTTATGCCCTAC	

The amplification products were analyzed on a 2.0% agarose gel (Tsingke, Beijing, China) in 1× TAE buffer, stained with GoldView (Yeasen Biotechnology, Shanghai, China), and visualized using the Gel Doc XR+ system (Bio‐Rad, Hercules, CA, USA).

### 2.8. Sensitivity and Specificity Assays

Bacterial samples spanning a range from 10^0^ to 10^5^ CFU or DNA quantities ranging from 1024 to 0.25 pg were employed as templates in the assay. The resulting PCR products were analyzed via gel electrophoresis to determine the lower detection limit. To evaluate the specificity of the mPCR assay and assess potential cross‐reactivity, genomic DNA from a panel of bacterial species was used as a template for amplification. The tested strains included *Streptococcus agalactiae*, *Streptococcus pluranimalium*, *Klebsiella pneumoniae*, *Streptococcus dysgalactiae*, *Enterococcus gallinarum*, *Enterococcus eurekensis*, *Globicatella sanguinis*, *Enterococcus cecorum*, *Streptococcus hyovaginalis*, and *S*. *suis*. Detailed information on these strains is provided in Supporting Information Table S3.

### 2.9. Ethical Statements

Animal experiments were carried out at the Laboratory Animal Center of Nanjing Agricultural University in accordance with the animal welfare guidelines of the Animal Research Committee of Jiangsu Province with the approval of the institutional ethics committee (Approval ID: NJAU.No20230301010).

## 3. Results

### 3.1. General Features of the *cps* Cluster of *S. pasteurianus*

**Figure 1:**
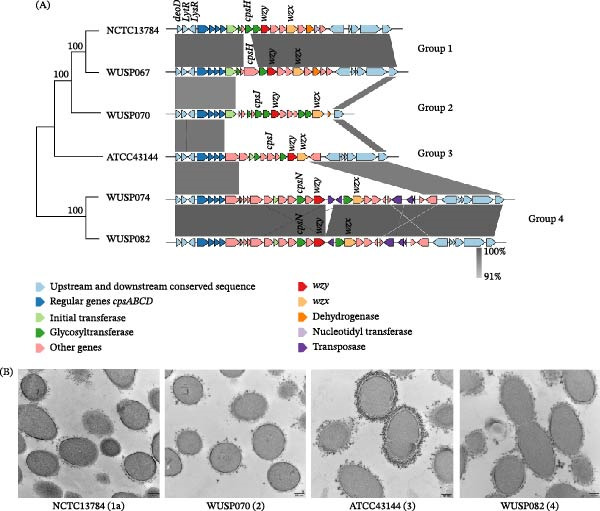
Genetic and phenotypic characterization of *S. pasteurianus* serotypes. (A) Organization of the *cps* clusters of representative *S. pasteurianus* strains. Predicted gene functions are indicated by different colors. Shaded regions denote genes whose deduced amino acid sequences share more than 91% identity. (B) TEM analysis showing the presence of capsules in representative strains of different *S. pasteurianus* serotypes.

Comparative genomic analysis of the 17 strains listed in Table 1 (the first dataset described in Section 2.1) classified the chromosomal *cps* loci into six distinct patterns (Figure 1A). In *S. pasteurianus*, the *cps* cluster is flanked by conserved genetic elements. Upstream, three conserved genes, *deoD*, *lytR*, and *lysR*, were identified, whereas a conserved downstream gene of ~1200 bp with an unknown function was also detected (Figure 1A). Moreover, within the *cps* cluster itself, the *cpsABCD* genes were found to be highly conserved across all *S. pasteurianus* strains analyzed, exhibiting >95% sequence similarity. All *cps* loci range from a minimum of 16,586 bp to a maximum of 30,975 bp in length, with the number of ORFs varying from 14 to 30 (Table 3). The G + C content percentage ranged from 32.18% to 34.97%.

**Table 3:** Representative *S. pasteurianus* strains used for *cps* locus analysis.

Strain	Accession number	Start	End	Size (bp)	Number of ORF	G + C content (%)
NCTC13784	NZ_LS483462.1	920,258	936,844	16,586	17	32.22
WUSP067	NZ_CP039457.1	884,069	901,590	17,521	17	32.18
WUSP070	NZ_CP116957.1	1,149,543	1,132,325	17,218	19	34.21
ATCC43144	NC_015600.1	843,890	858,285	14,395	14	34.97
WUSP074	NZ_CP116958.1	891,895	922,870	30,975	30	33.81
WUSP082	NZ_CP136943.1	1,380,977	1,351,154	29,823	28	33.65

Functional annotation of the *cps* cluster using Prokka and NCBI further revealed that genes encoding the initial glycosyltransferase, polysaccharide polymerase (Wzy), and flippase (Wzx), as well as those encoding other enzymes such as glycosyltransferases, acetyltransferases, transaminases, and modification enzymes, exhibited considerable variation across serotypes (Figure 1A). This finding suggests that CPS biosynthesis in *S. pasteurianus* serotypes occurs via the Wzx/Wzy‐dependent pathway.

### 3.2. The *cps* Cluster Grouping of *S. pasteurianus*

Phylogenetic analysis of the *cps* gene clusters from the 17 *S. pasteurianus* strains revealed four major groups (Figure 1A and Table 1). Group 1 comprised strains WUSP067, WUSP068, WUSP069, and WUSP084, which shared nearly identical capsular gene clusters (>99% sequence homology). Strain NCTC13784 also clustered within group 1 but harbored a variant form of the *cpsH* gene compared with the other four strains. Group 2 consisted solely of strain WUSP070. Group 3 included strains ATCC43144, WUSP083, WUSP085, WUSP086, WUSP087, and WUSP088, all of which exhibited highly conserved capsular gene clusters (>99% homology). Group 4 comprised strains WUSP074, WUSP079, WUSP080, and WUSP081, which also shared highly homologous capsular gene clusters (>99% homology); strain WUSP082 clustered within this group but lacked one gene present in the other four strains.

### 3.3. Serotype Determination of *S. pasteurianus*

Six representative strains (Figure 1A) were selected for the preparation of rabbit antisera. The results of the agglutination assays are summarized in Table 4. Antisera raised against group 1 strains exhibited agglutination titers greater than 1:32 with group 1 strains, whereas antisera against groups 2, 3, and 4 showed titers exceeding 1:64 with strains from their respective groups. Specifically, WUSP067 antiserum agglutinated strains WUSP068, WUSP069, WUSP084, and NCTC13784, and NCTC13784 antiserum agglutinated strains WUSP067, WUSP068, WUSP069, and WUSP084. No agglutination was observed between group 1 strains and antisera raised against strains from other groups, indicating that group 1 represents a distinct serotype, designated serotype 1. Similarly, strain WUSP070 (group 2) did not agglutinate with antisera against strains from other groups and was therefore classified as a distinct serotype, designated serotype 2. ATCC43144 antiserum agglutinated all five other strains within group 3, while no cross‐agglutination with strains from other groups was observed, demonstrating that these six strains constitute a third serotype, designated serotype 3. For group 4, agglutination was observed between the WUSP074 antiserum and strains WUSP079, WUSP080, WUSP081, and WUSP082, as well as between the WUSP082 antiserum and strains WUSP074, WUSP079, WUSP080, and WUSP081. No cross‐agglutination was detected with strains from other groups, confirming that these five strains belong to a fourth serotype, designated serotype 4.

**Table 4:** An agglutination reaction matrix comprising six bacterial strains.

Strains (group no.)	Anti‐NCTC13784	Anti‐WUSP067	Anti‐WUSP070	Anti‐ATCC43144	Anti‐WUSP074	Anti‐WUSP082
NCTC13784 (group 1)	+	+	−	−	−	−
WUSP067 (group 1)	+	+	−	−	−	−
WUSP070 (group 2)	−	−	+	−	−	−
ATCC43144 (group 3)	−	−	−	+	−	−
WUSP074 (group 4)	−	−	−	−	+	+
WUSP082 (group 4)	−	−	−	−	+	+

*Note:* “+” indicates a positive result in the slide agglutination test, and “−” indicates a negative result.

To determine whether serotypes 1 and 4 could be further subdivided, adsorption assays were performed using the corresponding antisera, followed by agglutination testing. As shown in Table 5, adsorption of the NCTC13784 antiserum with inactivated WUSP067 cells reduced its agglutination titer against WUSP067 to below 1:2, while a residual titer of 1:16 against NCTC13784 remained. This finding indicates that NCTC13784 and WUSP067 share common surface‐exposed capsular antigens but that NCTC13784 also expresses strain‐specific antigens absent from WUSP067. Accordingly, these strains were classified as distinct subtypes within serotype 1: NCTC13784 was designated serotype 1a and WUSP067 was designated serotype 1b.

**Table 5:** Cross‐adsorption agglutination assay of representative *S. pasteurianus* strains.

Antiserum	Absorbed with	Agglutination titer^a^	
		NCTC13784	WUSP067
Anti‐NCTC13784	Unabsorbed	1:32	1:16
	NCTC13784	<1:2	<1:2
	WUSP067	1:16	<1:2
Anti‐WUSP067	Unabsorbed	1:16	1:32
	NCTC13784	<1:2	<1:2
	WUSP067	<1:2	<1:2

Antiserum	Absorbed with	WUSP074	WUSP082
Anti‐WUSP074	Unabsorbed	1:64	1:64
	WUSP074	<1:2	<1:2
	WUSP082	<1:2	<1:2
Anti‐WUSP082	Unabsorbed	1:32	1:64
	WUSP074	<1:2	<1:2
	WUSP082	<1:2	<1:2

^a^Agglutination titers are expressed as the reciprocal of the highest antiserum dilution resulting in >90% visible agglutination.

In contrast, cross‐adsorption assays clarified the relationship between strains WUSP074 and WUSP082. Adsorption of WUSP074 antiserum with inactivated WUSP082 cells reduced its agglutination titer against WUSP074 to below 1:2, and similarly, adsorption of WUSP082 antiserum with inactivated WUSP074 cells reduced its agglutination titer against WUSP082 to below 1:2 (Table 5). These reciprocal results indicate that WUSP074 and WUSP082 share similar surface‐exposed capsular antigens and therefore belong to the same serotype, namely, serotype 4. In our previous study, we demonstrated that strain WUSP067 is encapsulated [9]. In the present study, representative strains of the remaining serotypes were further examined by TEM, and all exhibited a clear capsular layer (Figure 1B).

### 3.4. mPCR Assay for Serotype Identification

Serotype‐specific genes were identified through systematic analysis of *cps* clusters, with candidate genes requiring sequence similarity below 80% (Supporting Information Table S2). Based on standard primer design criteria, including amplicon size, compatibility of annealing temperatures, and sequence specificity, appropriate target genes were selected for the development of a mPCR assay. The selected targets included *DQN56_RS05055* (*cpsH*) from strain NCTC13784, *E8M05_RS04790* (*cpsH*) from strain WUSP067, *M0P24_RS05765* (*cpsJ*) from strain WUSP070, *SGPB_RS04335* (*cpsJ*) from strain ATCC43144, and *RJD36_RS07025* (*cpsO*) from strain WUSP082 (Supporting Information Table S4). These targets were combined to establish a single mPCR system capable of simultaneously distinguishing the five serotypes.

**Figure 2:**
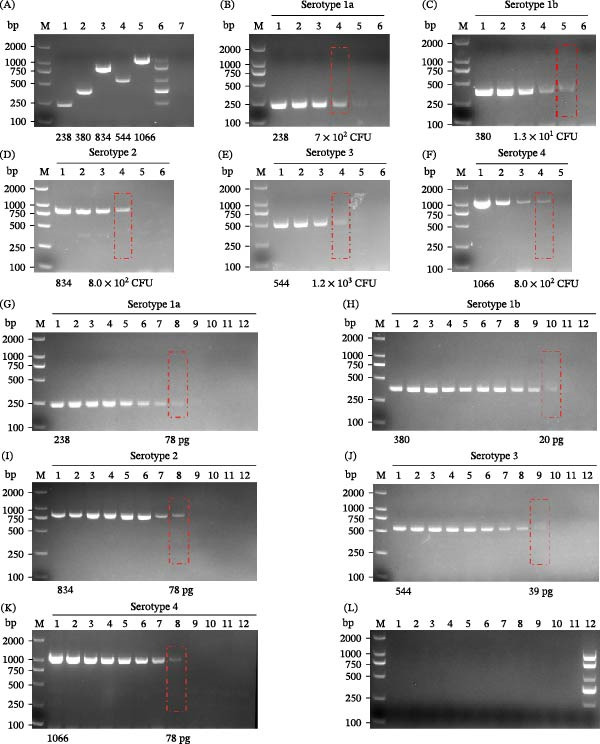
mPCR assay for serotyping *S*. *pasteurianus*. (A) Specificity of the mPCR assay using genomic DNA from individual strains or a mixed template. Lanes 1–5, strains NCTC13784 (serotype 1a, 238 bp), WUSP067 (serotype 1b, 380 bp), WUSP070 (serotype 2, 834 bp), ATCC43144 (serotype 3, 544 bp), and WUSP082 (serotype 4, 1066 bp), respectively; lane 6, mixed genomic DNA from the five strains; lane 7, negative control (H_2_O). M: DNA marker. (B–F) Sensitivity of the mPCR assay using serial dilutions of bacterial cultures. The numbers below the lanes indicate the detection limit (CFU). (B) NCTC13784: 7.0 × 10^2^ CFU; (C) WUSP067: 1.3 × 10^1^ CFU; (D) WUSP070: 8.0 × 10^2^ CFU; (E) ATCC43144: 1.2 × 10^3^ CFU; (F) WUSP082: 8.0 × 10^2^ CFU. (G–K) Sensitivity of the mPCR assay using serial dilutions of genomic DNA. The numbers below the lanes indicate the detection limit. (G) NCTC13784: 78 pg; (H) WUSP067: 20 pg; (I) WUSP070: 78 pg; (J) ATCC43144: 39 pg; (K) WUSP082: 78 pg. Lane 12 in each subpart represents the negative control (H_2_O). (L) Specificity of mPCR. Bacterial cultures used as templates: 1, *S. agalactiae*; 2, *S. pluranimalium*; 3, *K. pneumoniae*; 4, *S. dysgalactiae*; 5, *E. gallinarum*; 6, *E. eurekensis*; 7, *G. sanguinis*; 8, *E. cecorum*; 9, *S. hyovaginalis*; 10, *S. suis*; 11, negative control (H_2_O); 12, mixed DNA of the five *S. pasteurianus* serotype reference strains.

As shown in Figure 2A, following optimization of reaction conditions, the mPCR assay simultaneously amplified five distinct fragments from a mixture of genomic DNA derived from the five reference strains: a 238‐bp fragment (*cpsH*) from strain NCTC13784, a 380‐bp fragment (*cpsH*) from strain WUSP067, an 834‐bp fragment (*cpsJ*) from strain WUSP070, a 544‐bp fragment (*cpsJ*) from strain ATCC43144, and a 1066‐bp fragment (*cpsO*) from strain WUSP082. When genomic DNA from each individual strain was used as the template together with all five primer pairs, only a single serotype‐specific amplicon was detected, confirming the specificity of the assay (Figure 2A).

The sensitivity of the mPCR assay was evaluated using both bacterial cultures and purified genomic DNA from each representative strain. Detection limits using bacterial cultures as templates varied among the five serotypes and were determined to be 7.0 × 10^2^ CFU for strain NCTC13784 (Figure 2B), 1.3 × 10^1^ CFU for strain WUSP067 (Figure 2C), 8.0 × 10^2^ CFU for strain WUSP070 (Figure 2D), 1.2 × 10^3^ CFU for strain ATCC43144 (Figure 2E), and 8.0 × 10^2^ CFU for strain WUSP082 (Figure 2F). In terms of DNA quantity, the detection limits were 78 pg for strain NCTC13784 (Figure 2G), 20 pg for strain WUSP067 (Figure 2H), 78 pg for strain WUSP070 (Figure 2I), 39 pg for strain ATCC43144 (Figure 2J), and 78 pg for strain WUSP082 (Figure 2K).

To further assess the assay specificity and potential cross‐reactivity, genomic DNA from 10 additional bacterial species was tested. Only the mixed DNA from the five *S. pasteurianus* serotype reference strains yielded all five expected amplicons in the mPCR assay, whereas no amplification was observed for any of the other 10 bacterial species tested (Figure 2L).

Collectively, these results demonstrate that a sensitive and highly specific mPCR assay was successfully developed for identifying *S. pasteurianus* serotypes. This assay enables accurate serotype differentiation and provides a robust molecular tool for epidemiological investigations and diagnostic applications.

### 3.5. Serotyping of Clinical Strains and Publicly Available NCBI Strains

The serotypes of 18 clinical *S. pasteurianus* isolates from Thailand were determined by agglutination assays. Specifically, strain MN120 was classified as serotype 1a; strains MN93, MN104, MN121, MN177, MN231, and MN46 as serotype 1b; strain SKF5.1 as serotype 2; strains MN141, MN176, MN198, STC1034, STC1385, STC1394, and STC1049 as serotype 3; and strains MN106, STC126, and STC142 as serotype 4 (Table 1). Notably, the mPCR assay demonstrated complete concordance with the agglutination‐based serotyping results, yielding identical serotype assignments for all 18 isolates. This agreement confirms the accuracy and reliability of the mPCR assay as a rapid and reproducible molecular method for *S. pasteurianus* serotyping, effectively overcoming limitations associated with traditional antiserum‐based approaches.

To further explore the global distribution of *S. pasteurianus* serotypes, 31 publicly available genome sequences deposited in the NCBI database were analyzed based on *cps* cluster organization and serotype‐specific target genes (Supporting Information Table S1). This analysis assigned strains 1001285H and D12033531 to *cps* 1a; strains AM97‐57, D17293137, HC2909‐2, and S43 to *cps* 1b; strain UMB0765 to *cps* 2; strains 651SPAS, ERR10149241, ERR10960889, ERR1190541, S22, SGPN1, SGPN2, and SPSS39 to *cps* 3; and strains AF13‐3 and AF13‐35 to *cps* 4. The remaining 14 strains showed low similarity to the *cps* clusters of the five serotypes described above and were therefore considered nontypeable under the current serotyping scheme.

## 4. Discussion

Analysis of the *cps* cluster in *S. pasteurianus* revealed significant variability among different serotypes, while the locus was generally conserved within the same serotype. The G + C content of the *cps* locus (32.18%−34.97%) was lower than that of the whole genome (37%–37.5%), suggesting that the *cps* genes may have been acquired from an external source [19]. Comparative analysis of the *cps* cluster demonstrated substantial differences among serotypes, except for subtypes 1a and 1b, indicating significant variations in the composition of CPS across different serotypes of *S. pasteurianus*. Notably, serotypes 1a and 1b differ by only a single gene. In serotype 1a, *cpsH* (*DQN56_RS05055*) is predicted to encode a dTDP‐rhamnosyltransferase, whereas in serotype 1b, the *cpsH* (*E8M05_RS04790*) gene encodes a hypothetical protein. This finding suggests that subtypes 1a and 1b may share a similar CPS backbone structure but differ in their glycosyl composition, which could explain the observed antigenic cross‐reactivity between the two subtypes. These results underscore the role of glycosyltransferase specificity in determining the CPS structure and antigenic properties [20], providing critical insights into the molecular mechanisms underlying serotype differentiation in *S. pasteurianus*.

The *cps* cluster of *S. pasteurianus* serotype 4 strains is significantly larger than those of other serotypes and contains a substantial number of genes associated with complex polysaccharide metabolism and glycosyl modification. This expanded metabolic capacity may provide a significant advantage for survival and proliferation in diverse ecological environments [21]. In contrast, the *cps* clusters of other serotypes (e.g., serotypes 1, 2, and 3) are smaller and exhibit relatively limited metabolic capabilities. These differences may reflect the ecological pressures and adaptive selection faced by different serotypes during evolution [17, 22].

Comparative analysis revealed that the *cps* cluster of strains AM97‐57, D17293137, HC‐2909‐2, and S43 deposited in the NCBI database show high similarity to those of serotype 1b. With the exception of strain AM97‐57, for which the clinical status of the host is unclear, these strains were isolated from human cases presenting clinical symptoms. Together with the identification in our laboratory of four serotype 1b strains isolated from pigs with meningitis, these findings suggest that serotype 1b strains may be associated with an increased virulence potential, although further experimental validation is required.

Among the *S. pasteurianus* genomes currently available in the NCBI database (Supporting Information Table S1), nine strains originating from five countries (United States, South Korea, Kenya, China, and Bangladesh) exhibit *cps* clusters highly similar to those of serotype 3. Notably, serotype 3 displays both the broadest geographical distribution and the largest number of identified strains among the five serotypes, suggesting that it may represent the predominant circulating serotype of *S. pasteurianus* at the global level.

Human‐derived *S. pasteurianus* strains in this study were assigned to serotypes 1a, 1b, 3, and 4, indicating that multiple serotypes are involved in human infections. Given the limited number of isolates, the epidemiological significance of this distribution remains to be determined. Future studies with larger collections of clinical isolates from additional countries are warranted to further validate the serotype distribution, improve geographic representativeness, and clarify the prevalence and clinical relevance of different serotypes of *S. pasteurianus*.

The mPCR‐based serotyping method developed in this study provides a practical complement to conventional agglutination assays for *S. pasteurianus*. By targeting serotype‐specific variation within the *cps* locus, this assay enables the simultaneous detection of multiple serotypes in a single reaction and reduces the need for labor‐intensive antiserum production. Therefore, it may be useful for rapid molecular serotyping, particularly when specific antisera are unavailable or when large numbers of isolates need to be screened. In addition, this method can support the serotype assignment of archived isolates and the analysis of publicly available genomic datasets, thereby facilitating future studies on the serotype distribution and population structure of *S. pasteurianus*. Together with conventional serological methods, the mPCR assay provides a useful tool for epidemiological investigation and further studies of serotype‐associated biological differences in this pathogen.

## 5. Conclusion

In summary, we identified at least five distinct serotypes within *S. pasteurianus* and developed an mPCR assay for their molecular identification. These findings establish a serotyping framework for *S. pasteurianus* and provide a practical tool for future epidemiological studies, surveillance, and investigations of serotype‐associated differences in this pathogen.

## Author Contributions

Data curation: Shuoyue Wang, Xinchun Li, and Zongfu Wu. Formal analysis: Shuoyue Wang, Xinchun Li, Chenxu Zheng, Anusak Kerdsin, and Zongfu Wu. Investigation: Shuoyue Wang, Xinchun Li, Chenxu Zheng, and Zongfu Wu. Project administration: Zongfu Wu. Supervision: Zongfu Wu. Writing – original draft: Shuoyue Wang and Zongfu Wu. Writing – review and editing: Shuoyue Wang, Zongfu Wu, and Anusak Kerdsin.

## Funding

This work was supported by the National Key Research and Development Program of China (Grant 2023YFD1800503).

## Conflicts of Interest

The authors declare no conflicts of interest.

## Supporting Information

Additional supporting information can be found online in the Supporting Information section.

## Supporting information


**Supporting Information** Table S1: Serotype classification of *S. pasteurianus* genomes from NCBI. Table S2: Serotype‐specific genes. Table S3: Bacterial strains used in the PCR specificity assay. Table S4: Serotype‐specific target genes for mPCR assay.

## Data Availability

The draft genome sequencing data have been uploaded in the NCBI database under the accession numbers listed in Table 1. Additional supporting information can be found in the Supporting Information section of this article.

## References

[1] Gherardi G. , Palmieri C. , and Marini E. , et al.Identification, Antimicrobial Resistance and Molecular Characterization of the Human Emerging Pathogen *Streptococcus gallolyticus* Subsp. *pasteurianus* , Diagnostic Microbiology and Infectious Disease. (2016) 86, no. 4, 329–335, 10.1016/j.diagmicrobio.2016.09.019. 27720207

[2] Gray L. S. , Latorre J. D. , and Hernandez-Patlan D. , et al.Isolation, Characterization, and Experimental Infection of *Streptococcus gallolyticus* Subspecies *pasteurianus* From Commercial Turkeys With Acute Septicemia: A Pilot Study, Poultry Science. (2023) 102, no. 10, 10.1016/j.psj.2023.102950, 102950. PMC10407896 37540949

[3] Li M. , Gu C. , and Zhang W. , et al.Isolation and Characterization of *Streptococcus gallolyticus* Subsp. *pasteurianus* Causing Meningitis in Ducklings, Veterinary Microbiology. (2013) 162, no. 2–4, 930–936, 10.1016/j.vetmic.2012.11.038. 23294860

[4] Oliveira A. R. , de Castro M. F. , and Pimentel S. P. , et al. *Streptococcus pasteurianus*-Induced Valvular Endocarditis and Sepsis in a Puerperal Emperor Tamarin (*Saguinus imperator*), Journal of Medical Primatology. (2022) 51, no. 6, 388–391, 10.1111/jmp.12587. 35451506

[5] Floret N. , Bailly P. , and Thouverez M. , et al.A Cluster of Bloodstream Infections Caused by, *Streptococcus gallolyticus*, Subspecies, *pasteurianus*, That Involved 5 Preterm Neonates in a University Hospital during a 2-Month Period, Infection Control and Hospital Epidemiology. (2010) 31, no. 2, 194–196, 10.1086/650380. 20001733

[6] Hede S. V. , Olarte L. , Chandramohan L. , Kaplan S. L. , and Hulten K. G. , *Streptococcus gallolyticus* Subsp. *pasteurianus* Infection in Twin Infants, Journal of Clinical Microbiology. (2015) 53, no. 4, 1419–1422, 10.1128/JCM.02725-14. 25609731 PMC4365231

[7] Chand G. , Shamban L. , Forman A. , and Sinha P. , The Association of *Streptococcus gallolyticus subsp. pasteurianus*, Bacteremia With the Detection of Premalignant and Malignant Colonic Lesions, Case Reports in Gastrointestinal Medicine. (2016) 2016, 10.1155/2016/7815843, 7815843. 27555973 PMC4983331

[8] Sturt A. S. , Yang L. , Sandhu K. , Pei Z. , Cassai N. , and Blaser M. J. , *Streptococcus gallolyticus* Subspecies *pasteurianus* (Biotype II/2), a Newly Reported Cause of Adult Meningitis, Journal of Clinical Microbiology. (2010) 48, no. 6, 2247–2249, 10.1128/JCM.00081-10. 20357211 PMC2884481

[9] Wang S. Y. , Ma M. H. , and Liang Z. J. , et al.Pathogenic Investigations of *Streptococcus pasteurianus*, an Underreported Zoonotic Pathogen, Isolated From a Diseased Piglet With Meningitis, Transboundary and Emerging Diseases. (2022) 69, no. 5, 2609–2620, 10.1111/tbed.14413. 34871467

[10] Wang S. , Li X. , and Zheng C. , et al.Genomic Characteristics and Antimicrobial Resistance of the Underreported Zoonotic Pathogen *Streptococcus pasteurianus* and Its Co-Colonization With *Streptococcus suis* , Veterinary Microbiology. (2025) 303, 10.1016/j.vetmic.2025.110428, 110428. 39954531

[11] Ma M. , Wang S. , and Zhu X. , et al.The Identification of *Streptococcus pasteurianus* Obtained From Six Regions in China by Multiplex PCR Assay and the Characteristics of Pathogenicity and Antimicrobial Resistance of This Zoonotic Pathogen, Pathogens. (2023) 12, no. 4, 615. 37111501 10.3390/pathogens12040615 PMC10142533

[12] Geno K. A. , Gilbert G. L. , and Song J. Y. , et al.Pneumococcal Capsules and Their Types: Past, Present, and Future, Clinical Microbiology Reviews. (2015) 28, no. 3, 871–899, 10.1128/CMR.00024-15. 26085553 PMC4475641

[13] Whitfield C. , Wear S. S. , and Sande C. , Assembly of Bacterial Capsular Polysaccharides and Exopolysaccharides, Annual Review of Microbiology. (2020) 74, no. 1, 521–543, 10.1146/annurev-micro-011420-075607. 32680453

[14] Yother J. , Capsules of *Streptococcus pneumoniae* and Other Bacteria: Paradigms for Polysaccharide Biosynthesis and Regulation, Annual Review of Microbiology. (2011) 65, no. 1, 563–581, 10.1146/annurev.micro.62.081307.162944. 21721938

[15] Liu Z. , Zheng H. , and Gottschalk M. , et al.Development of Multiplex PCR Assays for the Identification of the 33 Serotypes of *Streptococcus suis* , PLoS ONE. (2013) 8, no. 8, 10.1371/journal.pone.0072070. PMC3739753 23951285

[16] Segura M. , Fittipaldi N. , Calzas C. , and Gottschalk M. , Critical *Streptococcus suis* Virulence Factors: Are They All Really Critical?, Trends in Microbiology. (2017) 25, no. 7, 585–599, 10.1016/j.tim.2017.02.005. 28274524

[17] Paton J. C. and Trappetti C. , *Streptococcus pneumoniae* Capsular Polysaccharide, Microbiology Spectrum. (2019) 7, no. 2, 10.1128/microbiolspec.GPP3-0019-2018, e00634-18. PMC11590643 30977464

[18] Elliott S. D. , Type and Group Polysaccharides of Group D Streptococci, The Journal of Experimental Medicine. (1960) 111, no. 5, 621–630, 10.1084/jem.111.5.621. 13726456 PMC2137285

[19] Nho S. W. , Hikima J. , and Park S. B. , et al.Comparative Genomic Characterization of Three *Streptococcus parauberis* Strains in Fish Pathogen, as Assessed by Wide-Genome Analyses, PLoS One. (2013) 8, no. 11, 10.1371/journal.pone.0080395, e80395. 24260382 PMC3832376

[20] Rini J. M. , Moremen K. W. , Davis B. G. , and Esko J. D. , et al. Varki A. , Cummings R. D. , and Esko J. D. , et al.Glycosyltransferases and Glycan-Processing Enzymes, Essentials of Glycobiology, 2022, Cold Spring Harbor Laboratory Press, 67–78.

[21] Kuznetsova E. , Proudfoot M. , and Gonzalez C. F. , et al.Genome-wide Analysis of Substrate Specificities of the *Escherichia coli* Haloacid Dehalogenase-like Phosphatase Family, Journal of Biological Chemistry. (2006) 281, no. 47, 36149–36161, 10.1074/jbc.M605449200. 16990279

[22] Croucher N. J. , Harris S. R. , and Fraser C. , et al.Rapid Pneumococcal Evolution in Response to Clinical Interventions, Science. (2011) 331, no. 6016, 430–434, 10.1126/science.1198545. 21273480 PMC3648787

