# Supplementary material for: Development of a Serotyping Scheme for Streptococcus pasteurianus: An Underreported Zoonotic Pathogen
Source: Transbound Emerg Dis. 2026 Jun 28;2026:6779233. doi: 10.1155/tbed/6779233 (PMC13311317; doi:10.1155/tbed/6779233)
Supplement: Supplementary file 1 — Supporting Information Table S1: Serotype classification of S. pasteurianus genomes from NCBI. Table S2: Serotype‐specific genes. Table S3: Bacterial strains used in the PCR specificity assay. Table S4: Serotype‐specific target genes for mPCR assay. [file TBED-2026-6779233-s001.docx]

**Supplementary Table 1. Serotype classification of *S. pasteurianus* genomes from NCBI**

| Serotype | Strains | Biological Sample | Country | Year | Isolation | Host | Health Status |
| --- | --- | --- | --- | --- | --- | --- | --- |
| 1a | 1001285H | SAMN15532893 | USA | Unknown | Feces | Human | Unknown |
|  | D12033531 | SAMN26135076 | Denmark | 2012 | Blood | Human | Diseased |
| 1b | AM97-57 | SAMN31809569 | China | 2014 | Feces | Human | Unknown |
|  | D17293137 | SAMN26135077 | Denmark | 2017 | Blood | Human | Diseased |
|  | HC-2909-2 | SAMEA3111506 | Belgium | Unknown | Blood | Human | Diseased |
|  | S43 | SAMN26876587 | USA | Unknown | Blood | Human | Diseased |
| 2 | UMB0765 | SAMN34996461 | USA | Unknown | Urine | Human | Diseased |
| 3 | 651_SPAS | SAMN03197853 | USA | Unknown | Unknown | Human | Unknown |
|  | ERR10149241 | SAMEA114096194 | South Korea | Unknown | Feces | Human | Healthy |
|  | ERR10960889 | SAMEA114061430 | Kenya | Unknown | Feces | Human | Healthy |
|  | ERR1190541 | SAMEA7846273 | China | Unknown | Gut | Human | Healthy |
|  | S22 | SAMN26876566 | USA | Unknown | Blood | Human | Diseased |
|  | SGPN1 | SAMN39700564 | USA | 2023 | Unknown | Unknown | Unknown |
|  | SGPN2 | SAMN39702318 | USA | 2023 | Unknown | Unknown | Unknown |
|  | SPSS39 | SAMEA5771531 | Bangladesh | Unknown | Gut | Human | Unknown |
| 4 | AF13-3 | SAMN31807845 | China | 2013 | Feces | Human | Unknown |
|  | AF13-35 | SAMN09734236 | China | 2013 | Feces | Human | Unknown |
| Nontypable | 100993126989 | SAMN26135075 | Denmark | 2010 | Blood | Human | Diseased |
|  | AF04-14B | SAMN31807704 | China | 2014 | Feces | Human | Unknown |
|  | AF04-26B | SAMN31807708 | China | 2014 | Feces | Human | Unknown |
|  | AL101002 | SAMN04074746 | China | 2010 | Brain | Duck | Diseased |
|  | AM109-09 | SAMN31808718 | China | 2014 | Feces | Unknown | Unknown |
|  | AM21-19 | SAMN09734891 | China | 2013 | Feces | Human | Unknown |
|  | ERR1606358 | SAMEA110405862 | China | Unknown | Feces | Pig | Diseased |
|  | GED7275A | SAMN03956210 | Unknown | Unknown | Vagina | Human | Unknown |
|  | HGUT-00246 | SAMEA5849748 | China | Unknown | Gut | Human | Unknown |
|  | S37 | SAMN26876581 | USA | Unknown | Blood | Human | Diseased |
|  | SRR16280113 | SAMEA114122822 | China | Unknown | Feces | Human | Unknown |
|  | SRR17798009 | SAMEA114065786 | China | Unknown | Feces | Human | Diseased |
|  | UMB3102 | SAMN35153926 | USA | Unknown | Urine | Human | Diseased |
|  | UMB8624A | SAMN34996657 | USA | Unknown | Urine | Human | Diseased |

**Supplementary Table 2. Serotype-specific genes**

| Strain (serotype) | ID | Predicted products | Size (bp) |
| --- | --- | --- | --- |
| NCTC13784 (1a) | DQN56_RS05055 | glycosyltransferase | 933 |
| WUSP067 (1b) | E8M05_RS04790 | glycosyltransferase | 1920 |
| WUSP070 (2) | M0P24_RS05785 | dTDP-4-dehydrorhamnose 3,5-epimerase family protein | 427 |
|  | M0P24_RS05780 | DUF1972 domain-containing protein | 504 |
|  | M0P24_RS05775 | glycosyltransferase | 477 |
|  | M0P24_RS05770 | glycosyltransferase | 498 |
|  | M0P24_RS05765 | glycosyltransferase | 972 |
|  | M0P24_RS05760 | glycosyltransferase | 1044 |
|  | M0P24_RS05755 | polysaccharide polymerase | 1167 |
|  | M0P24_RS05750 | glycosyltransferase | 894 |
|  | M0P24_RS05745 | NAD-dependent epimerase/dehydratase family protein | 840 |
|  | M0P24_RS05740 | acyltransferase | 633 |
|  | M0P24_RS05735 | glycosyltransferase | 933 |
|  | M0P24_RS05730 | glycosyltransferase | 858 |
|  | M0P24_RS05725 | flippase | 1542 |
|  | M0P24_RS05720 | transposase | 321 |
| ATCC43144 (3) | SGPB_RS04320 | DegT/DnrJ/EryC1/StrS family aminotransferase | 1227 |
|  | SGPB_RS04325 | glycosyltransferase | 693 |
|  | SGPB_RS04330 | glycosyltransferase | 771 |
|  | SGPB_RS04335 | ATP-grasp fold amidoligase family protein | 903 |
|  | SGPB_RS04340 | glycosyltransferase | 1032 |
|  | SGPB_RS04345 | AAA family ATPase | 633 |
|  | SGPB_RS04350 | glycosyltransferase | 843 |
|  | SGPB_RS04355 | polysaccharide polymerase | 1272 |
|  | SGPB_RS04360 | flippase | 1434 |
| WUSP082 (4) | RJD36_RS06965 | polysaccharide biosynthesis C-terminal domain-containing protein | 666 |
|  | RJD36_RS06970 | transposase | 1106 |
|  | RJD36_RS06975 | transposase | 1257 |
|  | RJD36_RS06980 | transposase | 462 |
|  | RJD36_RS06985 | hypothetical protein | 189 |
|  | RJD36_RS06990 | non-hydrolyzing UDP-N-acetylglucosamine 2-epimerase | 1119 |
|  | RJD36_RS06995 | acyltransferase family protein | 1002 |
|  | RJD36_RS07000 | lipopolysaccharide biosynthesis protein | 1488 |
|  | RJD36_RS07005 | flippase | 1191 |
|  | RJD36_RS07010 | transposase | 771 |
|  | RJD36_RS07015 | polysaccharide polymerase | 1440 |
|  | RJD36_RS07020 | acyltransferase family protein | 999 |
|  | RJD36_RS07025 | glycosyltransferase | 1164 |
|  | RJD36_RS07030 | glycosyltransferase | 1143 |
|  | RJD36_RS07035 | ATP-grasp domain-containing protein | 1197 |
|  | RJD36_RS07040 | glycosyltransferase | 663 |
|  | RJD36_RS07045 | DegT/DnrJ/EryC1/StrS family aminotransferase | 1203 |
|  | RJD36_RS07050 | polysaccharide biosynthesis protein | 1599 |
|  | RJD36_RS07055 | acylphosphatase | 375 |
|  | RJD36_RS07060 | LbetaH domain-containing protein | 462 |
|  | RJD36_RS07065 | hypothetical protein | 237 |
|  | RJD36_RS07070 | hypothetical protein | 162 |

**Supplementary Table 3. Bacterial strains used in the PCR specificity assay**

| **Strain** | **Species** | **Geographic origin** | **Isolation Site** | **Host** | **Health Status** |
| --- | --- | --- | --- | --- | --- |
| GD201008-001 | *Streptococcus agalactiae* | Guangzhou | Brain | Tilapia | Diseased |
| WUQT025 | *Streptococcus pluranimalium* | Hunan | Lung | Pig | Diseased |
| WUQT019 | *Klebsiella pneumoniae* | Jiangsu | Tonsil | Pig | Healthy |
| WUQT020 | *Streptococcus dysgalactiae* | Jiangsu | Tonsil | Pig | Healthy |
| WUQT027 | *Enterococcus gallinarum* | Hunan | Lung | Pig | Diseased |
| WUQT037 | *Enterococcus eurekensis* | Hunan | Lung | Pig | Diseased |
| WUQT036 | *Globicatella sanguinis* | Hunan | Lung | Pig | Diseased |
| WUQT028 | *Enterococcus cecorum* | Hunan | Lung | Pig | Diseased |
| WUQT033 | *Streptococcus hyovaginalis* | Hunan | Lung | Pig | Healthy |
| GZ0565 | *Streptococcus suis* | Guangzhou | Brain | Pig | Diseased |

**Supplementary Table 4. Serotype-specific target genes for mPCR assay**

| Serotype 1a | Target gene: *DQN56_RS05055* (*cpsH*) |
| --- | --- |
| atggttaaaatagcagctggtatcgtactgtataatcctgataatattgagcgacttaatcactgtattcagagtattttaccacaagtttcgaaagtatacatttatgataatagttcgcaaaaatatgattattcttttacagaaaatgttatctataaatctagaggaaaaaatctagggatagcgtttgctttaaatgaattgatgcaaatggctgaagcagatgaggttgagtggttaataacattggatcaagattctattgtcccagataatttagtccaacattatttaactactctttatgaagaaaaagacaataatgtagcgataatttgtcctcaagttattgataaaaggcgtaaatacatgcatgttcaaacttctaaaaagatggaatatgttgatgaatgtattacatcagctagttgtacgtctgtagaggcttggaaaaaagtaggtggttttgacaactggctttttattgatttagttgataatgaattttgtaaaagattagttgtttctgattataagattttaagattaaattttttagtattagatcaagaatttggtaacatcgaacctaaatcagaacttgaacaaaaattttggatttttttaagcaaagtgtttagaaatcaaaatattgcgaaattatcatataaaaaaaatgttagtccaatgcgagtttattatacttgtcgaaacattatttatgttaataaaaaattaagaaattatgaaaaaacagcttattcaaattatcattgcaaaggttactttggatttatcatcgcttttattttgccaagttttcttagagctaaaaataaatggttagtgttaaaagaaattattagaggaacaaaagatggtttaaattcagccataatagaatggcatccatcaaaaatggacaagtaa | |
| Serotype 1b | Target gene: *E8M05_RS04790* (*cpsH*) |
| atgaagatagttaataagataaaacatacactaaatactggaaagcctattgattcacaaaaatttatcttatctttccaagattataaggtagtatcttttgatatttttgataccttgctaaagcgtaatgttgcaaaaccgacagatgtatttgcttatatagaacaaaaatttggcatagaaaattttaagcaaaaaagaattgaagctgagagaatagctcgaagccagtcatttgattcagaaatttcattggtggacatctataatcagtatggaatagattattcaaaggtagaactgcaggcagaatcagaactgttaactttaaatcaagatatgtttttggttttccaagaagcagtaaaaaataaaacagtcattctaacatcagacatgtatctacctgagacatttattgtagatattttaaatcgagagggcattactggctatcataaattatatttatcatcaactgttggtgttacaaaaagcaatggtaaaatttttgatttaatcattgaagatttatgtattaatcgtgcagatatcatccatattggagattcatttcattcagactacaatatacctagaaaaaaaggaatagccgcaaagcatttaccaactactattaagaaatcacactatcgactatccggtgatgctattgagattaatattatcaatagttttattaataattctatacctagcactgcggattcttattatagatttggctatgaaaaatttggaatgtttttatggggattttctaaatggctgcatgaatcgttactaaatgaaaatattcaaaaggtttattttttttcacgagatggtttaatcatgaaacaagcctttgatatgctttttgatgacattgaaacccattatttagaagtatctagaagggctttaagggttccgattttatggaaaaattattctcttgatcatgttattgatatgatatctccctctaagatgatatccttatcctcagtgtttgatggtctgggtttaaatatgcatgactacacagatttattgactacgtataactactcttttgagacttattttgatcgtaaagaattggtcaataataaaaaattcgcagagttttatcaagaactatcagcagatattgaaagtaactctcgtaatgagtatgacttattagtgaaataccttgatcagcatcatatcgaaggaaagtttgcaattgtagatattggatggtcaggtggcatgcaacgctacttaaaagaaaccttggatacattaaaaataagcaataatattacaggatactatattggtattgctgattattataaacgaaatgtaaaagcagttccttctttaaatttaaagggttatttgtttgatttttccaagaatacaacagaaattgataaacgtaggccctttgtcggcttatttgaatctctttttttagagcaagatggttctgttgaaaaatatattaatgataatggtgtgataaaagcaaaaagattttcatatgaatattttgaaaacggaaaaccaactgaggagtttcaagctattaaaaaattacaaagaggagctttggattttgtcaaaagttttggtaatccatcaattgaaatatctgcagatcaattattttatggtctggagcaaacgggtctttttcctaacaaacaagatattaaattatttgcggactttcgattttttgatgatggagaaatcaattatcttgccaaacctcagcatttattaacctacttgctacatattagacaattgaaaaaagattttttaatgtgccgttggaaaattggttttttaaagcgattattaaagattaaacttccttatcaaactatttacaattatctatccaaatataaataa | |
| Serotype 2 | Target gene: *M0P24_RS05765* (*cpsJ*) |
| ttaatcatataggcctaaatcaaaaatatattcgtcaacgtattttccgccattgagggcgtagctgaatttagatccttgcgttatggaactaattaaatatttacatctggaaagcagaatcatttttactaagtatttctgtccacgaagttttttgttatcctccaagacattcgacctgctcagaacgtctttgccatcgtaatcatagataaatgaatcatacgaaacgattttgatgtgctctccaaacttatttcttaaagcatcataaatatttccatcttctgtaactaaaaaaacggaggcatgatattgtctgataaagttttcaatttgttgttccacttgcttcacatttggctgtacatattcaccggaaggacgcaattttatataatccgttcccctaacgtaaacgccaatgcaattatttatatcaagagcctttgcttccttttctacaagatttaaaacttcatcactaaaacttaaattcttgtttaaaagctcatagctcattttttcaagttttgtattaaaaaaaatctccgattcaaacagtcccaaaggattaacatcttttaatgtccaaccggaaagatagacatttttactattgtaaatttcctcttctttaatgtcggagggttgtttgaaaaaatagttccaaacattattttttccgtcataatattgtgttttatatttcttccaatcaacgaacggaatgtacccatttttttttgcataatctatgtatatgacctgtttgtgaatcagtgacagcaagccttccacgccatcttggtaatccggtttaacaatataaattattttgtcattattacattttccatagctcttctttttctgaagaatatttcttctcgcaatgaaataataaatatattttaacttgtatcctaaaaatctatgctctctttgaaatttcaaacctactggcat | |
| Serotype 3 | Target gene: *SGPB_RS04335* (*cpsI*) |
| atggaaagaaacagcgttgcgtataaactacgcagaaaagggcaagcaatagcatttactttattaagtcatgaaacattatctaaattatattatagaatatgtttgcataagaaactaaacttaaagaatcctaaaacatttaatgaaaaattacagtggtataaactttattattgtgcagataaccaattgatcgttgattgtactgataagtatcatgtaagagactatttagttaataaaggttatcaaaatttattgactggtcttctaggggtatgggataatgcagaagatattgattgggaatcattgcccaatcaatttgtaatgaagtgtacgcatgggtgtgcatataatattatttgtactgataaaacaacgtttgattttgctaaagcaacaaaacagttaaataactggttgagagaagatttctcgagattcaatgttgaacttcattatggaaaagtaaaacccaaaataatctgtgaagaatttcttggagaggcaattactgactataagttcttttgttttaatggaaatccagagtttttttatgtatcaactgatttggtaaatgatcgtcaggcggaaatggctttttttgatatggatggttcaaaaattccactagttagagaagattacaaggatattggagaagtaatttttccagattatttaaatgatatgattgaggtatcaaaaaaactctctagcgactttccttttgtgagagttgacttctttattacgaatgactcatttaaatttgcagagttgacttttaccccaggagcagggatgataccgattaatccaatagaatatgatatagaatggggtaaaaaatttaaactacctaagcatgattatcaatatggaaggatgtaa | |
| Serotype 4 | Target gene: *RJD36_RS07025* (*cpsN*) |
| atgaaaaaaatattagcatttaatgattactatattccagcaataaaatgtggaggacctgtaacgagtattaataatgctgtaaatgcattaaaagatgaatatgaattttatattgaagcagttaatcatgattttggagataaaacaccatttccaggaataggtgataaatggtatactgtaggagcggcacatgtcagatatcataaagatggtgaattggattttaattataaaaaaatggaagaatttattgaagaagtaaatccagatttaatgtggttttcagggcttctcgtaccaaataaaatacataatgcaatacgtgttggtgaaaaaagggggattcctgttgtgatttcaccacgaggagaagctagtccagatagaatgtgcttaaagggctacaaaaaatatccatatgcagctttagtttcaatattaggtatatataaaaagaaaaatgttttttttcatgtaacaagcgatgatgagagtgtaggattaaaaaaatattttcatattaatgaagaaaaaataactaaagtaccaaatataggaattatgccatcattgagagaagatagatatagtaaaaaagcaggaacagtacgtgcaatgtttatatcgagaattcatgaagtaaaaaatcttgactatgcaattaaagttttttcaaaaattaaagagcaaggcgagtttgatatttatgggccaatagagtcaaaagattattggaataagtgcgtagaattaataaagaaagttccagataacattacaataaaatattgtgggtgtattaatccgaatgaagttagtaaaatatatagtcaatatgattgctttttatttccaacacaaaatgaaaattatggacatgtaatagcagaagcattagctaatcaatgtcctgttatattgagtcggggaacgacgccatgggatgatattgatgaaaaagcaggatttgtttgtaaattatataatgatgatgagtttataaatgcattatcaaagatagctgcgatgaatgcaaaagaatatgaaaatctgatggaaaaaacacatttgtattatgttgaaaaaaaaaaagaatcagaggcaatagtagggcataaagctatgtttgagtcgataataaagaccatgattaaatga | |
